# Is neurogenic inflammation involved in tendinopathy? A systematic review

**DOI:** 10.1136/bmjsem-2022-001494

**Published:** 2023-02-09

**Authors:** Shimon Vinay Zedeck Wasker, Dimitris Challoumas, Wai Weng, George A C Murrell, Neal L Millar

**Affiliations:** 1 Orthopaedic Research Institute, St George Hospital Sydney, University of New South Wales, Sydney, New South Wales, Australia; 2 School of Infection and Immunity, College of Medicine, Veterinary and Life Sciences, University of Glasgow, Glasgow, UK

**Keywords:** Tendon, Tendinopathy, Immunology

## Abstract

Neurogenic pain and inflammation have been hypothesised to play an important role in tendinopathy. This systematic review aimed to present and assess the evidence on neurogenic inflammation in tendinopathy. A systematic search was conducted through multiple databases to identify human case–control studies assessing neurogenic inflammation through the upregulation of relevant cells, receptors, markers and mediators. A newly devised tool was used for the methodological quality assessment of studies. Results were pooled based on the cell/receptor/marker/mediator assessed. A total of 31 case–control studies were eligible for inclusion. The tendinopathic tissue was obtained from Achilles (n=11), patellar (n=8), extensor carpi radialis brevis (n=4), rotator cuff (n=4), distal biceps (n=3) and gluteal (n=1) tendons. Through pooling the results of included studies based on the marker of neurogenic inflammation assessed, we identified possible upregulation of protein gene product 9.5 (PGP 9.5), N-methyl-D-aspartate Receptors, glutamate, glutamate receptors (mGLUT), neuropeptide Y (NPY) and adrenoreceptors in tendinopathic tissue versus control. Calcitonin gene-related peptide (CGRP) was not found to be upregulated, and the evidence was conflicting for several other markers. These findings show the involvement of the glutaminergic and sympathetic nervous systems and the upregulation of nerve ingrowth markers supporting the concept that neurogenic inflammation plays a role in tendinopathy.

**WHAT IS ALREADY KNOWN ON THIS TOPIC**

- The pathophysiology of tendinopathy remains incompletely understood.
- Neurogenic inflammation is assumed to play a role in tendinopathy.
- Better understanding of the implicated pathophysiology mechanisms can help with treatment of this challenging condition.

**WHAT THIS STUDY ADDS**

- We confirmed that neurogenic inflammation is present in tendinopathic tissues.
- Our findings demonstrated involvement of the glutaminergic and sympathetic nervous systems in tendinopathy.
- Nerve ingrowth markers were also found to be upregulated in diseased versus healthy tendon tissues.

**HOW THIS STUDY MIGHT AFFECT RESEARCH, PRACTICE OR POLICY**

- These findings suggest that further investigation on the role of neurogenic inflammation is warranted, partcilary to trty and address pain mechanisims in tendinopathy.

## Introduction

Tendinopathy is a common, often disabling condition associated with tendon pain, functional decline and reduced exercise tolerance.^1–3^ Physical examination may encompass local swelling, tenderness and decreased range of motion. Some patients experience sudden ruptures without any pre-existing clinical symptoms, suggesting that tendinopathy development may, in some cases, be asymptomatic.^4^ Histopathological evaluation of tendinopathic biopsies shows disorganised and calcified collagen fibres, elevated ground substance levels, morphological alterations of mitochondria and nuclei and the presence of mucoid patches, vacuoles and lipid cells.^4–9^

It has been hypothesised that tendinopathy occurs when tendon tissue undergoes chronic overload, which induces a state of hyperthermia, hypoxia and reduced vascularity, such that it cannot recover adequately.^10^ Individuals’ variations (age, genetics, sports activities, environmental conditions) may account for different repair threshold-associated responses to overload.^4^ The contemporary ‘biochemical’ tendon pain model hypothesised that an unidentified biochemical mediator-driven stimulation of nociceptors in or around the tendon was the cause of pain in tendinopathy.^11^ Furthermore, it has been suggested that tendinopathic damage occurs within an asymptomatic and symptomatic phase due to imbalanced protective and regenerative processes that ensue as part of a response to tendon overuse.^2^ The continuum model of tendinopathy described chronic tendon disease as three continuous stages^11^: stage 1 is when tenocytes develop a homogeneous, non-inflammatory metaplastic and proliferative cell response to load bearing; stage 2 comprises a healing response mediated by chondrocytes and myofibroblasts which secrete proteoglycan and collagen; stage 3 includes tenocyte apoptosis, and matrix and collagen breakdown, with no scope of reversibility.^11^ In light of growing evidence, the ‘biochemical’ hypothesis has been revived in recent literature, suggesting that locally produced substances might drive vascular regulation, tissue modulation and/or pain mediation.^12^

Neurogenic inflammation is a subtype of inflammation that occurs when peripheral terminals of primary sensory neurons are triggered by local depolarisation, axonal reflexes or dorsal root reflexes, such as in the event of mechanical stress or injury.^13^ These peripheral terminals release bioactive substances, such as substance P and calcitonin gene-related peptide (CGRP), which trigger the ‘classical/chemical’ inflammatory pathway upstream.^13^ Neuromediators play an essential role in maintaining tendon homoeostasis.^14^ It has been reported that tendinopathic pain is accompanied by neovascularisation, neoinnervation and elevated ‘algogenic’ substances (CGRP, glutamate, substance P), which have been hypothesised to cause neurogenic inflammation.^14–19^ Finally, among others, the involvement of catecholamines, neurokinin-1 receptors (NK-1R) and N-methyl-D-aspartate receptors (NMDR-1) has been reported in tendinopathy.^19–26^

Our study aimed to review, assess and present the current evidence regarding neurogenic inflammation in tendinopathy. This may potentially provide further insights into the pathophysiology of this multifaceted and debilitating disorder and allow us to discover new therapies.

## Materials and methods

This systematic review was conducted according to the Preferred Reporting Items for Systematic Reviews and Meta-Analyses statement.

### Search strategy

A systematic search was undertaken in November 2021 via the following databases: CINAHL PLUS EbscoHost, EMBASE, Medline OVID, Scopus, SPORTDiscus and Web of Science. The following Boolean operators were used: Tendinopathy OR Tendino* OR Tendinitis OR Tendonitis OR Tendon rupture OR Ruptured Tendon AND Neurogenic inflammat* OR Neurogenic-mediated inflammat* OR Neuro-mediated inflammat* OR Neuroinflammat* OR Neuro-inflammat* OR neur* OR nerv*.

For databases that use medical subject headings (MeSH) [AMED, CINAHL, EMBASE, MEDLINE and SPORTDiscus], free terms for the Neurogenic Inflammation (NI) were combined with the subject heading “nervous system” using the Boolean operator “OR”. This method was also used for free terms and subject headings related to tendinopathy. These two groups (NI and tendinopathy) were then joined using the Boolean operator “AND”. Only free terms were used for databases that did not use relevant subject headings (Biological Abstracts, Scopus and Web of Science). Review articles were used to identify eligible articles missed in the initial search. Additionally, reference list screening and citation tracking in Google Scholar were performed for each relevant article.

### Inclusion and exclusion criteria

Papers were included only if they were clinical case–control studies in humans (including those that obtained sampling of tendons for analyses) investigating the presence of neurogenic inflammation in tendinopathic tendons through the presence of cells, receptors, markers and mediators relevant to neurogenic inflammation. Eligible participants were of any age presenting with a clinical diagnosis of tendinopathy or spontaneous tendon rupture, considering the assumption that predominantly tendinopathic tendons are prone to spontaneous ruptures.^27^ Diagnostic criteria of tendinopathy included a clinical presentation of chronic pain or loss of function of the affected tendon, with or without confirmatory imaging. We only included case–control studies and not other types of observational studies as we deemed it important to assess the presence of neurogenic inflammation markers in tendinopathic tissue compared with healthy tissue, as some of these may be present in both and be irrelevant.

Studies were excluded if they only assessed paratendinous tissue, were in vitro studies wherein tissue or cells were treated with cytokines or other agents or modified, animal studies, reviews, case reports or case series and studies that could not be obtained in English.

The search, selection of studies and data analysis were performed independently by two authors (SVZW and WW). Agreement on inclusion was achieved after a review of the full-text articles and a joint decision by both authors based on the inclusion/exclusion criteria. Data were then extracted using a spreadsheet that included patient demographics, symptom duration, investigations, control group type, tissue analysis method, statistical methods and methodological characteristics.

### Quality assessment

Methodological quality was assessed using a 15-point scale. This quality assessment tool was constructed using a set of questions assimilated by the authors from several sources.^20–23^ It was designed so that each question would clearly and unambiguously target one important source of bias (online supplemental table 1). The first five questions (Q1–5) focus on the recruitment method employed in the studies. Questions 6–8 and 13 evaluate the relevance of each study in furthering our knowledge about neurogenic inflammation in tendinopathy. Questions 9–12, 14 and 15 assess whether the methodology employed in each study is valid and minimises any risk of bias. Studies were deemed as ‘high quality’ (>12), ‘moderate quality’ (10–12), or ‘low quality’ (<10) based on their overall score in the study quality assessment tool.

10.1136/bmjsem-2022-001494.supp1 Supplementary data

Each article was independently evaluated by two authors (SVZW and WW). Where disagreements existed, the opinion of a third author (DC) was sought, and a consensus was reached among the three assessors.

### Data handling

Data were extracted from each of the included papers by two of the authors (SVZW and WW) and were tabulated to facilitate analysis. The results of studies assessing the presence/upregulation/involvement of specific markers of neurogenic inflammation were pooled on a binary scale (upregulated or not upregulated), and an overall (pooled) result was obtained for each one of these markers. The three possible results for each marker were ‘upregulated’, ‘not upregulated’ or ‘unclear due to conflicting evidence’. The overall result for each marker was derived from an agreement between the two first authors and the decision was based on the number of studies demonstrating a positive versus a negative outcome and the quality of these studies as assessed using our devised quality assessment tool. In the absence of a clear majority of either ‘upregulated’ or ‘not upregulated’, the overall result was deemed as ‘unclear’ (due to conflicting evidence). Only markers assessed by three or more studies were used for pooling. No meta-analyses were performed.

## Results

### Search yield

**Figure 1:**
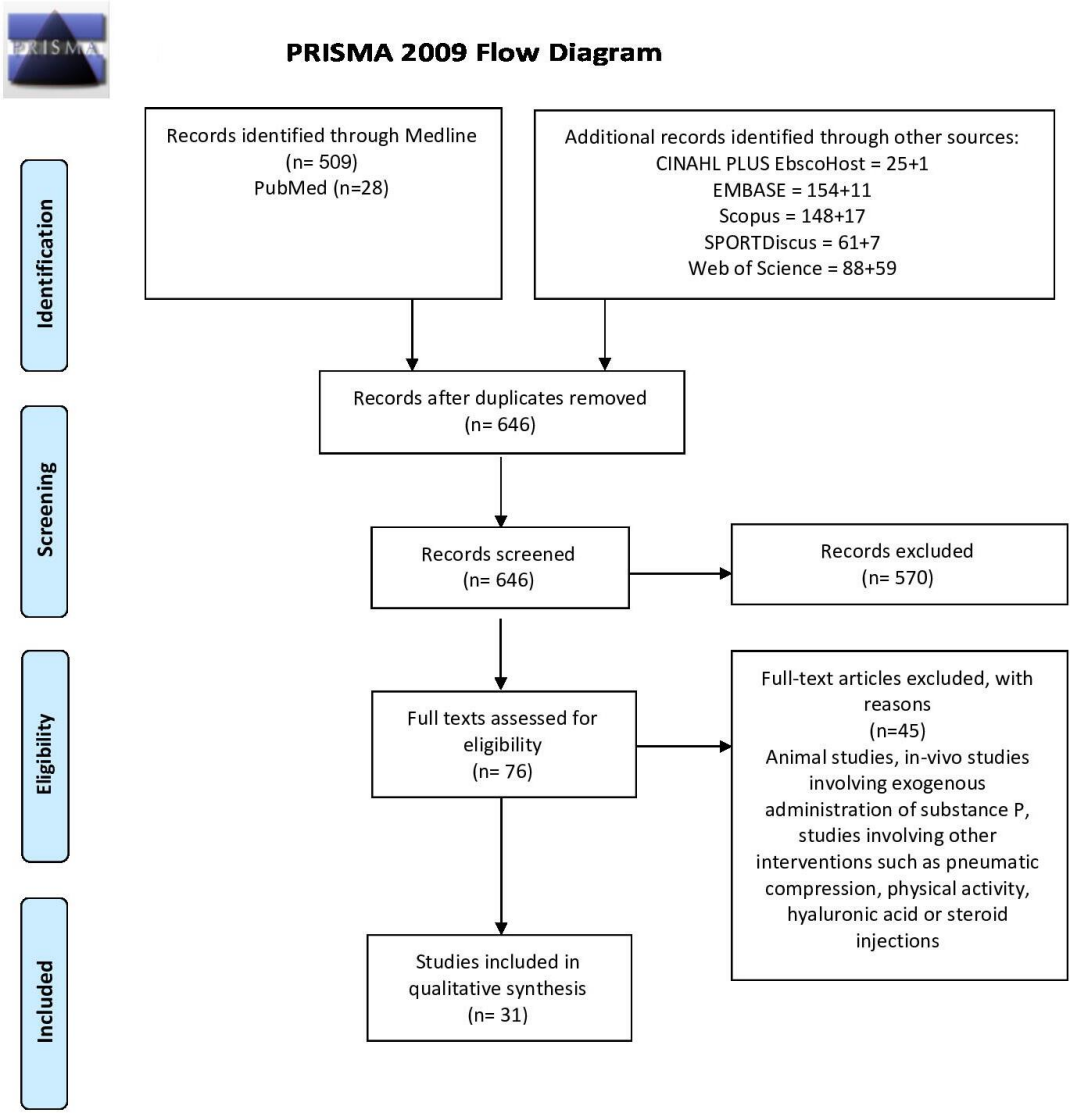
Preferred Reporting Items for Systematic Reviews and Meta-Analyses (PRISMA) flow diagram of included studies. Adapted from: Moher D *et al*.^56^

The search of the 6 databases yielded 646 papers. On eliminating duplicates and irrelevant articles and those that did not match the inclusion and exclusion criteria, 31 case–control studies were found to be eligible for inclusion (figure 1).

Online supplemental tables 2 and 3 show the most important characteristics of the included studies. The findings of each study are summarised in online supplemental tables 4 and 5, illustrating the relevant markers of neurogenic inflammation assessed in each study.

### Study characteristics

Of the 31 studies included, 10 were related to the Achilles tendon, 7 to the patellar tendon, 2 both to Achilles and patellar tendons, 4 to the rotator cuff tendon, 4 to the extensor carpi radialis brevis (ECRB) tendon, 3 to the bicep tendon and 1 to the gluteal tendon. The included studies related to patients with painful tendinopathy, and all studies stated specific diagnostic criteria. Samples were obtained during surgery for 23 studies, while the remaining 8 performed direct biopsy sampling. The mean ages of the overall patient groups were as follows: 44 years (Achilles), 26 years (patellar), 53 (rotator cuff), 44 years (ECRB) and 62 years (biceps). The control group consisted of healthy asymptomatic patients in 30 studies and cadaveric control in one study.

### Quality assessment

Five studies were deemed as ‘high quality’, 10 as ‘moderate quality’ and 16 as ‘low quality’. The results of the study quality assessment are shown in online supplemental table 6.

### Main findings

The results are presented below based on the neurogenic inflammation marker assessed (table 1). Online supplemental table 5 summarises the findings of each study and shows the pooled result for each neurogenic inflammation marker separately.

**Table 1:** Overall result for each marker of neurogenic inflammation occurring from qualitative pooling of the results of all studies that assessed them

Marker	Study	Result	Overall result
AChE	Alfredson *et al*, 2000a^41^	↑	Unclear
	Alfredson *et al*, 2001b^39^	↑	
	Danielson *et al*, 2006^57^	↔	
Adrenoreceptors	Franklin *et al*, 2014^25^	↑	Involved
	Tosounidis *et al*, 2013^45^	↑	
	Bjur *et al*, 2008^46^	↑	
	Danielson *et al*, 2007b^47^	↑	
CGRP	Sahmey *et al*, 2016^27^	↑	Unclear
	Bjur *et al*, 2005^31^	↑	
	Sahmey *et al*, 2016^27^	↑	
	Franklin *et al*, 2014^25^	↔	
	Sasaki *et al*, 2013^28^	↔	
	Singaraju *et al*, 2008^34^	↔	
	Danielson *et al*, 2007 (2)^30^	↔	
Glutamate	Schizas *et al*, 2010^26^	↑	Involved
	Alfredson *et al*, 2001^39^	↑	
	Alfredson *et al*, 2000a^41^	↑	
	Alfredson *et al*, 2000b^41^	↑	
	Alfredson *et al*, 1999^40^	↑	
	Dean *et al*, 2015^24^	↔	
mGLUT receptors	Dean *et al*, 2015^24^	↑	Involved
	Franklin *et al*, 2014^25^	↑	
	Schizas *et al*, 2012^36^	↑	
	Scott et al, 2008^42^	↑	
Neuropeptide Y	Sasaki *et al*, 2013^28^	↑	Involved
	Tosounidis *et al*, 2013^45^	↑	
	Bjur *et al*, 2009^49^	↑	
	Bjur *et al*, 2008^46^	↑	
	Danielson *et al*, 2007b^47^	↑	
NK-1R	Andersson *et al*, 2008^35^	↑	Unclear
	Forsgren *et al*, 2005^44^	↑	
	Franklin *et al*, 2014^25^	↔	
NMDAR	Franklin *et al*, 2014^25^	↑	Involved
	Schizas *et al*, 2012^36^	↑	
	Schizas *et al*, 2010^26^	↑	
	Alfredson *et al*, 2001^39^	↑	
	Alfredson *et al*, 2000 (1)^41^	↑	
	Dean *et al*, 2015^24^	↔	
PGP 9.5	Sahemey *et al*, 2016^27^	↑	Involved
	Dean *et al*, 2015^24^	↑	
	Franklin *et al*, 2014^25^	↑	
	Sasaki *et al*, 2013^28^	↑	
	Schizas *et al*, 2012^36^	↑	
	Xu *et al*, 2011^15^	↑	
	Danielson *et al*, 2007b^47^	↑	
	Lian *et al*, 2006^29^	↑	
	Bjur *et al*, 2005^31^	↑	
Substance P	Sahmey *et al*, 2016^27^	↑	Unclear
	Christensen *et al* 2015^32^	↑	
	Fearon *et al*, 2014^33^	↔	
	Franklin *et al*, 2014^25^	↔	
	Sasaki *et al*, 2013^28^	↔	
	Singaraju *et al*, 2008^34^	↔	
	Danielson *et al*, 2007^30^	↔	
	Schizas *et al*, 2012^36^	↑	
	Andersson *et al*, 2008^35^	↑	
	Lian *et al*, 2006^29^	↑	
	Bjur *et al*, 2005^31^	↑	
	Ljung *et al*, 1999^37^	↑	
Tyrosine hydroxylase	Zeisig*et al*, 2009^48^	↑	
	Bjur *et al*, 2008^46^	↑	
	Danielson *et al*, 2007a^30^	↑	Unclear
	Danielson *et al*, 2007b^47^	↑	
	Franklin *et al*, 2014^25^	↔	
	Lian *et al*, 2006^29^	↔	

↑, upregulated; ↔, not upregulated.

AChE, Acetylcholinesterase; CGRP, calcitonin gene-related peptide; mGLUT, metabotrophic glutamate receptor; NMDAR, N-methyl-D-aspartate receptor; PGP 9.5, protein gene product 9.5.

#### Protein gene product 9.5

A total of nine case–control studies assessed for the involvement of protein gene product 9.5 (PGP 9.5) in tendinopathy. Four were of high quality,^15 24–26^ three of moderate quality^28–30^ and two of low quality.^31 32^ Based on the pooled findings of these studies, PGP 9.5 is likely to be upregulated in tendinopathy.

#### Substance P

A total of 12 case–control studies assessed the involvement of substance P in tendinopathy. Four^25 26 33 34^ were of high, four^27–29 34^ of moderate and four^9 30 35 36^ of low quality. While seven of these studies^26 28 30 32 33 37 38^ suggest an upregulation of substance P, the other five studies^25 29 31 34 35^ demonstrate no difference; therefore, the overall result is unclear due to conflicting evidence

#### Calcitonin gene-related peptide

Seven case–control studies assessed the involvement of CGRP in tendinopathy; one^25^ was of high, three^28 29 34^ of moderate and three^31 32 35 38^ of low quality. Three of the studies^27 31 37^ found upregulation of CGRP in tendinopathy, and four^26 29 31 35^ found no differences. The overall result is, therefore, unclear due to conflicting evidence.

#### Glutamate

A total of six case–control studies assessed the involvement of glutamate in tendinopathy. Four^39–42^ were of low, one^26^ of moderate and one^24^ of high quality. Based on the pooled findings of these studies, glutamate is likely to be upregulated in tendinopathy.

#### Glutamate receptor: mGLUT (metabotrophic glutamate receptor)

A total of four case–control studies^24–26 36^ investigated the involvement of glutamate receptors (mGLUT) in tendinopathy, and all four demonstrated the upregulation of these receptors. Three^26 32 33^ were of high and one^43^ of low quality. mGLUT receptors are, therefore, likely to be upregulated in tendinopathy.

#### N-methyl-D-aspartate Receptors

A total of six case–control studies assessed the involvement of NMDAR in tendinopathy. Three^24–26 44^ were of high, one^32^ of moderate and two^38 39^ of low quality. Based on the pooled findings of these studies, NMDAR is likely to be upregulated in tendinopathy.

#### Neurokinin 1 receptor

Three case–control studies assessed the involvement of NK-1R in tendinopathy. Two^37 45^ were of low and one^25^ of high quality. The overall result is unclear due to conflicting evidence.

#### Adrenoreceptors

A total of four case–control studies assessed the involvement of adrenoreceptors in tendinopathy. Two^25 46^ were of high and two,^31 47^ were of low quality. Based on the pooled findings of these studies, adrenoreceptors are likely to be upregulated in tendinopathy.

#### Tyrosine hydroxylase

A total of seven case–control studies assessed for the involvement of tyrosine hydroxylase in tendinopathy. Three^31 47 48^ were of low, two^30 49^ of moderate and one^25^ of high quality. Based on the pooled findings of these studies, the overall result is unclear due to conflicting evidence.

#### Neuropeptide Y

A total of five case–control studies assessed for the involvement of neuropeptide Y in tendinopathy. Three^47^ were of low, one of moderate and one^46^ of high quality. Based on the pooled findings of these studies, neuropeptide Y is likely to be upregulated in tendinopathy.

#### Acetylcholinesterase

Three case–control studies assessed the involvement of AChE in tendinopathy. All three^39 40 50^ of these were of low quality. Based on the pooled findings of the studies, the overall result is unclear due to conflicting evidence, as two showed upregulation of AChE and one no difference.

## Discussion

**Figure 2:**
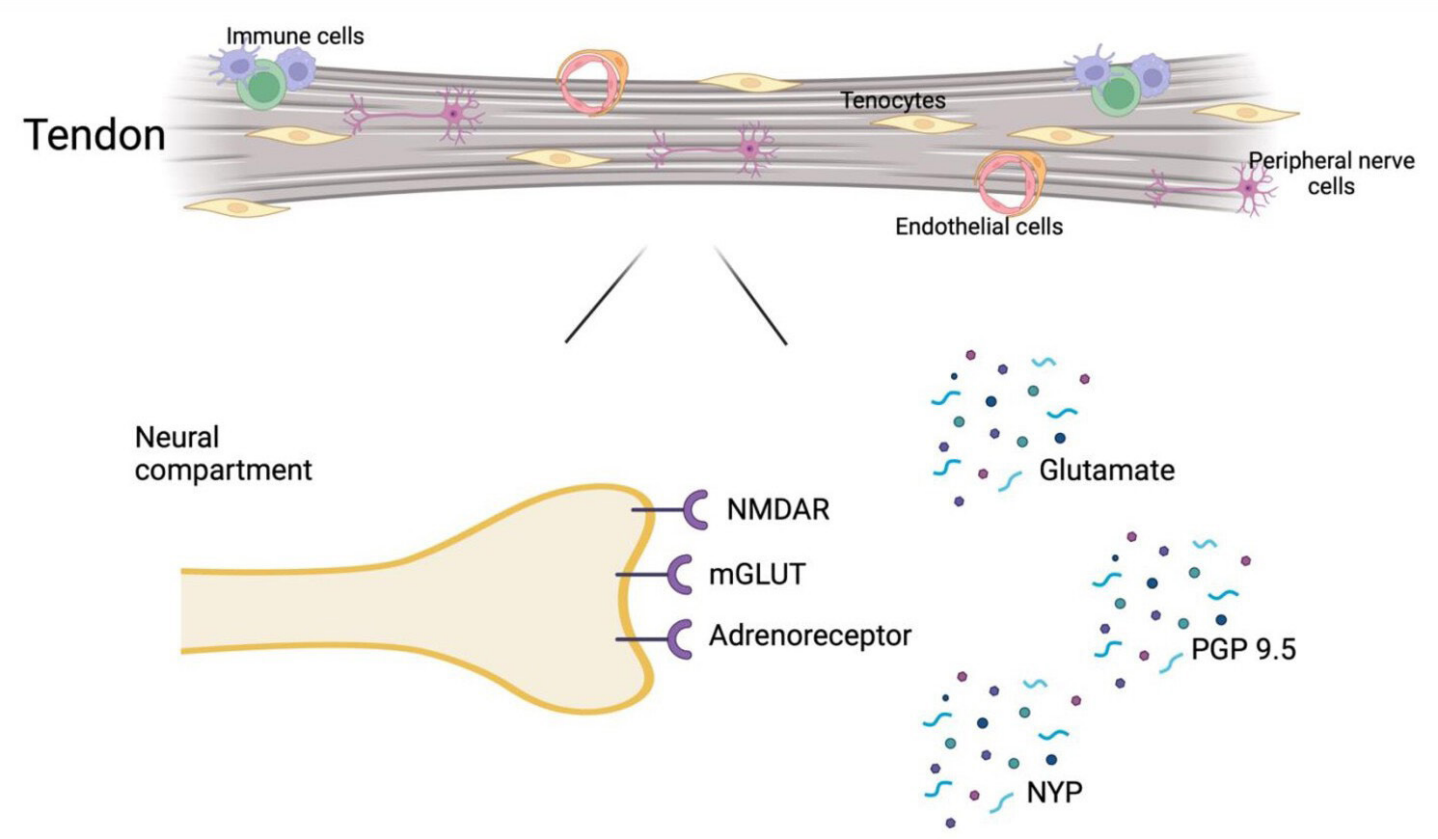
Mediators of neurogenic inflammation involved in tendinopathy. The neural compartment within the tendon detailing the mediators discovered through the systematic review. In the homoeostatic state the neural compartment plays a role in proprioception. It interacts with immune cells to modulate adaptive responses in the normal tendon, but excessive stimulation leads to tissue breakdown, degeneration and neoinnervation involving the glutamatergic and autonomic systems. The systematic review found N-methyl-D-aspartate receptors (NMDAR), adrenoreceptors and glutamate receptors (mGLUT) to be upregulated in tendinopathic tissues. Furthermore the release of neuropeptides, such as neuropeptide Y (NPY), glutamate and protein gene product 9.5 (Pgp 9.5) stimulates immune cell activation, releasing various agents, which modulate a variety of cell activities in the matrix.

The purpose of our study was to summarise the evidence for neurogenic inflammation in tendinopathy. We found six neuronal markers that are likely upregulated in tendinopathic samples versus control. These were PGP 9.5, NMDAR, glutamate, glutamate receptors (mGLUT), neuropeptide Y (NPY) and adrenoreceptors. Of the remaining markers, CGRP was shown not likely to be involved. However, there was conflicting evidence regarding the involvement of substance P, NK-1R, tyrosine hydroxylase and AChE in tendinopathy. These findings suggest the likely involvement of the glutaminergic (glutamate, NMDAR, mGLUT) and sympathetic nervous (NPY, adrenoreceptors) systems and the upregulation of nerve ingrowth markers (PGP 9.5) (figure 2). These results derived from pooling of studies of different tendinopathy locations, which may involve different pathophysiological processes and neuronal markers, therefore making definitive conclusions is difficult.

Our findings are partly consistent with two previously conducted reviews. Jewson *et al*^22^ mainly investigated the involvement of the sympathetic nervous system in tendon disease; they included 13 observational studies (including cohort studies without controls) and concluded that sympathetic innervation (adrenoreceptors α1 or α2A and β1, NPY, tyrosine hydroxylase) is likely not upregulated in tendon proper but may be upregulated in paratendinous tissues in patients with tendinopathy.^22^ The review by Dean *et al*^23^ evaluated the correlation between pain symptoms and the trend in peripheral neural markers in painful human tendinopathy. They concluded that painful tendinopathy is accompanied by an upregulation of nerve ingrowth markers (PGP9.5, GAP43) and glutaminergic system (Glutamate, NMDAR, mGlut receptors). Specifically, substance P was particularly implicated in rotator cuff tendinopathy. This latter study was very similar to ours in that it only included case–control studies and assessed the presence of neurogenic inflammation in general; we added four studies published after the review and presented updated results, having handled data slightly differently. Similarly to Dean *et al*, we found strong evidence for the upregulation of the glutaminergic system and nerve ingrowth markers in tendinopathic tissue. However, our results were unclear regarding the upregulation of substance P. In contrast to the conclusions of Jewson *et al*, we found that the sympathetic nervous system is likely to be upregulated in tendinopathic tissue.

Neurogenic inflammation is mediated by the peripheral nervous system responding to noxious stimuli.^13^ These stimuli include signals associated with tissue damage (ATP, uric acid and hydroxynonenals), environmental signals (heat, acidity and chemicals), pathogen-associated signals (bacterial or viral proteins), as well as chemokines released from immune cells.^13^ These signals are detected by various receptors such as danger-associated molecular pattern receptors (TRP channels, P2X channels), pattern recognition receptors (Toll-like receptors and Nod-like receptors) and cytokine receptors, which are present on afferent neurons. Nociceptive stimulation of sensory neurons generates antidromic axon reflexes that cause the release of neuropeptides. These neuropeptides trigger an inflammatory response, including recruitment and activation of immune cells, vasodilation and exudation.^13^

Ackermann. discussed the growing evidence for the role of neural elements in tissue homoeostasis and healing in connective tissues such as tendons and ligaments.^14 51^ Several studies have consistently demonstrated positive immunohistochemical staining for the protein marker PGP 9.5 in tendinopathy.^24 29^ PGP 9.5 stains for all nerves and was particularly noted to be upregulated in abnormal tenocytes and perivascular areas of the tendon sample.^24 26 34 35 37^ Xu *et al* hypothesised that this association between neoinnervation and angiogenesis may be involved in pain signalling in tendinopathy.^15^ Sahmey *et al* suggested that in tendinopathy, tenocytes behave like neuroendocrine cells and secrete peptides such as substance P, CGRP and VEGF, which trigger an inflammatory cascade of events downstream.^27^ The predominant proportion of upregulated innervation in tendinopathic samples corresponded to sympathetic innervation, evidenced by positive NPY staining.^29 31 32 46 47^ Sasaki *et al* suggested that NPY may reflect central sensitisation secondary to nascent sympathetic innervation. However, only a very small proportion of tendon tissue staining was associated with sensory innervation, evidenced by decreased expression of CGRP and substance P.^29^ This is consistent with the findings of this systematic review, where the overall result indicates that CGRP appears not to be upregulated in tendinopathy. Sasaki *et al* and Lian *et al* suggested that the loss of sensory innervation of the tendinosis tissue and the upregulation of sympathetic innervation are crucial in understanding chronic tendon pathology.^28 29^ Lian *et al* observed the sprouting of sensory nerve endings inside the tendon properly and suggested that it reflects the intensification of nociceptive signalling secondary to recurring mechanical impetus. They further propose that the upregulation of sympathetic innervation may very well act contrary to nociceptive signalling, thus helping to modulate and reduce tendon pain.^30^

The autonomic nervous system is largely involved in regulating blood flow to the tendons during exercise, wherein acetylcholine causes vasodilation, while sympathetic neuropeptides mediate vasoconstriction.^52^ Danielson *et al* reported the presence of alpha1-adrenoreceptor and tyrosine hydroxylase in tendinopathic tendons and therefore hypothesised the local catecholamine synthesis in tendinopathy.^30^ Furthermore, the same group notes that adrenergic receptors stimulation produces degenerative/apoptotic events and cell proliferation, which is known to be present in the early and late phases of tendinopathy.^31 53^ They also demonstrated the presence of muscarinic receptors, choline acetyltransferase and vesicular acetylcholine transporters in tendinopathic tissue samples, which suggests an upregulation of the cholinergic system as part of the neurogenic inflammatory response in tendon disease.^50^

This systematic review suggests that the overall result concerning the upregulation of substance P in tendinopathy is conflicting. Murphy and Hart noted that substance P altered the expression of plasminogen activator and plasminogen activator inhibitor in the ligament, epiligament and synovial tissues of rabbits.^53^ Han *et al*^54^ observed higher substance P gene expression levels in human tendinopathic tissue compared with healthy tenocytes. Furthermore, they demonstrated that exposing healthy tenocytes to substance P resulted in increased cellular proliferation, synthesis of type 3 collagen and morphological alteration similar to what we see in tendinopathic tenocytes.^55^ Burssens *et al*^55^ reported exogenous substance P injection to induce fibroblast proliferation and improved collagen organisation in injured rat Achilles’ tendon.

Several studies have confirmed glutamate, an excitatory neuropeptide, to be upregulated in tendinopathy.^26 39–41^ Additionally, glutamate receptors such as NMDAR and mGLUT have also been identified and localised in tendinopathic tissue samples.^26 36 39^ These changes were prominent in morphologically altered tenocytes and vasculature and were absent in control samples. A possible explanation of glutamate upregulation may be its role in cell-hyperexcitation, pain signalling and cell proliferation/differentiation.^39 51^ Franklin *et al* suggested that the early inflammatory changes in tendinopathy upregulate the expression of glutaminergic receptors, which in turn results in peripheral sensitisation.^25^

This review also accounts for other neural markers, which may be implicated in tendinopathy’s pathophysiology and clinical presentation. These include neuron-specific enolase, PAR receptors, KA1, Nav1.7, TRPA1 BDKRB2, S-100, BDNF, CB1, GAP 43, NGF, BDNF P75, M2 Ach Receptor, ChAT, VAChT. However, a comprehensive analysis of their upregulation was not possible due to limited studies undertaken on these specific markers.

We recognise the limitations of our review. Results were pooled without accounting for the location of tendinopathy, assuming that the potential upregulation of neurogenic inflammation markers would be consistent in all tendinopathies; subgroup analyses would result in fewer studies being pooled, which could compromise the strength of evidence. However, to the best of our knowledge, we conducted a detailed literature search and included all eligible studies and performed a thorough study quality assessment, which was accounted for in our overall results.

## Conclusion

We found strong evidence for the upregulation of nerve ingrowth markers, the glutaminergic and sympathetic nervous systems in tendinopathic tissue. The involvement of the parasympathetic nervous system and the upregulation of sensory nerves remains unclear. More high-quality case–control studies are needed to contribute data to future reviews that will hopefully report results with higher strength of evidence and clarify the possible involvement of markers for which evidence was conflicting.
