# Supplementary material for: Is neurogenic inflammation involved in tendinopathy? A systematic review
Source: BMJ Open Sport Exerc Med. 2023 Feb 9;9(1):e001494. doi: 10.1136/bmjsem-2022-001494 (PMC9923261; doi:10.1136/bmjsem-2022-001494)
Supplement: Supplementary data [file bmjsem-2022-001494supp001.pdf]

BMJ Publishing Group Limited (BMJ) disclaims all liability and responsibility arising from any reliance placed on this supplemental material which has been supplied by the author(s)

Supplementary Information: Neurogenic Inflammation in Tendinopathy

Number	Criteria	Decision Rule	Yes/No/Unclear
1	**Recruitment** method clearly reported?	Yes, if the study states how participants were recruited.<br><br>No, if the method of recruitment is not stated or is unclear.	
2	**Inclusion/exclusion criteria** clearly described?	Yes, if clear eligibility criteria for participant inclusion and/or exclusion are reported.<br><br>No, if eligibility criteria are not given or are unclear	
3	**Study Population:** Are the **cases and controls** recruited over the same period of time?	Yes, if it is stated that cases and controls were recruited concurrently.<br><br>No, if cases and controls were not recruited concurrently, if recruitment times were unclear or if recruitment times were not reported.<br>Score N/A if only one group.	
4	**Study Population:** Are cases and controls drawn from the same population?	Yes, if both the case and control group were drawn from the same source population.<br><br>No, if case and controls groups are from different populations or if unclear.<br>Score N/A if only one group.	
5	**Study Population:** Are the participants representative of the population from which they were recruited?	Yes, if the study states that consecutive eligible participants were used, participants were randomly selected, or all participants were used from the source population.	

6	**Case**: Is the case definition explicit?	Yes, if the criteria for diagnosing injury is clearly described. OR<br>Yes, if diagnosis was made using established criteria and an appropriate reference is given (e.g. a consensus document).<br><br>No, if the criteria for diagnosis are not given or are unclear.<br>N/A if no case group.	
7	**Control:** Is the control group free from injury?	Yes, if the method of confirming that the control group is free from the target injury is reported.<br><br>No, if the method of confirming the control group is free of injury is not given or is unclear.<br>Score N/A if only case group.	
8	**Method**: Were markers of neurogenic inflammation assessed identically in the case and control group?	Yes, if the measurement of neurogenic inflammation was stated to be identical in the case and control group.<br><br>No, if there were any differences in measurement technique between the case and control group.<br>N/A if only one group.	
9	**Method:** Was the reliability of the measurement technique reported?	Yes, if reliability estimates of the measurement technique was calculated or a reliability study was cited.	
10	**Method:** Was assessor blinding reported?	Yes, if is stated that the assessor measuring sympathetic involvement was blind to injury status.	

		No, if the assessor is aware of injury status or if no mention is made of assessor blinding.<br>N/A if only one group.	
11	**Method:** Were the observational tests used to assess the main outcomes appropriate?	Yes, if the observational tests used were appropriate for the research question and the data with minimum 2 independent observers?<br><br>No, if no quantitative or semi-quantitative descriptive analysis was reported.	
12	**Method:** Are the distributions of principal confounders in each group of subjects to be compared clearly described?	Yes, if summaries of participant age, sex, BMI (or height and weight) are provided for the case and control group.<br><br>No if study did not provide data for at least these factors.	
13	**Data Analysis:** Are differences between neurogenic inflammation markers between the case and control group clearly reported?	Yes, if the comparison of neurogenic inflammation between case and control groups is clearly described. OR<br>Yes, if data is provided in sufficient detail to calculate a comparison between the case and control groups<br><br>No, if comparison is not clearly described.<br>No, if comparison is given as significant or non-significant without p-value or detailed data.<br>N/A if only one group	
14	**Data Analysis:** Does the study provide estimates of the random variability in the data for the main outcomes?	Yes, if an estimate of data variability is provided for sympathetic involvement. Acceptable estimates include SD, SE, and IQR.	

		No, if an estimate of data variability is not provided.<br>Range of scores not acceptable.<br>N/A if no statistical analysis performed.	
15.	**Study Design:** Study limitations addressed?	Yes, if study limitations appropriately addressed.<br><br>No, if study limitations are not listed.	

Supplementary Table 1: Methodological Quality Assessment Tool. *N/A, Not applicable; SD, standard deviation; SE, standard error of the mean; IQR, Interquartile range*

BMJ Publishing Group Limited (BMJ) disclaims all liability and responsibility arising from any reliance placed on this supplemental material which has been supplied by the author(s)

S.No.	Study	Type of study	Specimen	Tendon	Tissue	Detection
**1.**	Schmalzl J et al., 2019	Case-Control	Biopsies from living tissue	Long head of biceps tendon	Tendon stump	Immunohistochemistry
**2.**	Sahmey et al 2016	Case Control	Biopsies from living tissue	Supraspinatus tendon	Tendon and tendon sheath	Immunohistochemistry
**3.**	Christensen J et al 2015	Case-Control	Biopsies from living tissue	Achilles tendon	Mid-portion of Achilles Tendon (from ventral side)	Immunofluorescence
**4.**	Dean et al 2015	Case control	Biopsies from living tissue	Supraspinatus tendon	Within 1cm of bony insertion into greater tuberosity	Immunohistochemistry
**5.**	Fearon et al 2014	Case Control	Biopsies from living tissue	Greater trochanteric bursa and gluteal tendon	Mid tendon	Immunohistochemistry
**6.**	Franklin et al 2014	Case Control	Biopsies from living tissue	Rotator Cuff tendon	Supraspinatus tendon	Immunohistochemistry
**7.**	Sasaki et. al., 2013	Case Control	Biopsies from living tissue	Extensor Carpi Radialis Brevis	Capsular aspect of ECRB tendon	Immunohistochemistry
**8.**	Tosounidis et al 2013	Case Control	Biopsies from living tissue and cadavers	Long head of Bicpes Brachii (LHB) tendon	Tendon proper and surrounding tissue	Immunohistochemistry
**9.**	Schizas et al 2012	Case Control	Biopsies from living tissue	Patellar tendon	Tendon proper and peritendinous loose CT	Immunohistochemistry
**10.**	Bagge et al 2012	Case Control	Biopsies from living tissue	Achilles tendon	Mid-portion	In Situ Hybridization and Immunohistochemistry

**11.**	Bjorklund et al 2011	Case Control	Biopsies from living tissue	Achilles tendon	Tendon mid-portion (tendon proper and paratendinous connective tissue)	Immunofluorescence
**12.**	Xu et al 2011	Case Control	Biopsies from living tissue	Rotator Cuff tendon - torn Supraspinatus tendon and matched intact Subscapularis tendon	Torn edges of Supraspinatus and intact subscapularis tendons	Immunohistochemistry
**13.**	Schizas et al 2010	Case Control	Biopsies from living tissue	Patellar tendon		Immunofluorescence
**14.**	Bagge et al 2009	Case Control	Biopsies from living tissue	Achilles tendon	Ventral part of mid tendon (tendon mid-portion)	Immunofluorescence
**15.**	Bjur et al 2009	Case Control	Biopsies from living tissue	Achilles tendon	Mid portion + paratendinous tissue	Immunohistochemistry
**16.**	Zeisiget al 2009	Case Control	Biopsies from living tissue	Extensor carpi radialis brevis (ECRB) tendon	Muscle origin at the lateral epicondyle (TE) & origin of the flexor muscles at the medial	Immunofluorescence

					epicondyle (GE)	
**17.**	Singaraju et al 2008	Case Control	Biopsies from living tissue and cadavers	Long head of the biceps brachii (LHBB) tendon	Portion of the LHBB tendon above the bicipital groove	Immunohistochemistry
**18.**	Andersson et al 2008	Case Control	Biopsies from living tissue	Achilles tendon	Mid-portion tendon	Immunofluorescence & In Situ Hybridization
**19.**	Bjur et al 2008	Case Control	Biopsies from living tissue	Achilles tendon	Mid-portion tendon	Immunofluorescence & In Situ Hybridization
**20.**	Scott et al 2007	Case Control	Biopsies from living tissue	Patellar and Achilles tendon		Immunofluorescence & In-Situ Hybridisation
**21.**	Danielson et al 2007 (1)	Case Control	Biopsies from living tissue	Patellar tendon		In-Situ Hybridisation
**22.**	Danielson et al 2007 (2)	Case Control	Biopsies from living tissue	Patellar tendon	Tendon proper	Immunofluorescence
**23.**	Danielson et al 2006	Case control	Biopsies from living tissue	Patellar tendon	Proximal patellar tendon	Immunofluorescence & Immunostaining using EnVision detection
**24.**	Lian et al 2006	Case Control	Biopsies from living tissue	Patellar tendon		Immunofluorescence
**25.**	Bjur et al 2005	Case Control	Biopsies from living tissue	Achilles tendon	Mid-portion tendon	Immunohistochemistry

**26.**	Forsgren et al 2005	Case control	Biopsies from living tissue	Patellar or Achilles tendon	Proximal part of Patellar tendon Mid-portion of Achilles tendon	Immunofluorescence
**27.**	Alfredson et al 2001 (1)	Case Control	Biopsies from living tissue	Achilles tendon		Microdialysis – high performance liquid chromatography and Immunohistochemistry
**28.**	Alfredson et al 2001 (2)	Case Control	Biopsies from living tissue	Patellar tendon		Microdialysis- high performance liquid chromatography and Immunohistochemistry
**29.**	Alfredson et al 2000	Case Control	Biopsies from living tissue	Extensor Carpi Radialis Brevis tendon		In situ microdialysis
**30.**	Alfredson et al 1999	Case control	Biopsies from living tissue	Achilles tendon		In situ microdialysis
**31.**	Ljung et al 1999	Case Control	Biopsies from living tissue	Extensor Carpi radialis brevis tendon	Dorsal aspect of tendon insertion	Immunohistochemistry

Supplementary Table 2: Study Characteristics – part A

BMJ Publishing Group Limited (BMJ) disclaims all liability and responsibility arising from any reliance placed on this supplemental material which has been supplied by the author(s)

**Study**	**Group**	**Tendon Status**	**Sample Size**	**Sex**	**Age, years (mean and/or range)**
1. Schmalzl J et al., 2019	Case (arthroplasty, rotator cuff surgery, Isolated biceps surgery)	Tendinopathy	11	9 M<br>2 F	60-82
	Control (arthroplasty, rotator cuff surgery, Isolated biceps surgery)	No Tendinopathy	11	4 M<br>7 F	46-67
2. Sahmey et al 2016	Case (rotator cuff surgery)	Tendinopathy	4	2 M<br>2 F	39-53
	Control (arthroscopic re-stabilisation)	No Tendinopathy	1	1 M	20
3. Christensen et al., 2015	Case (Achilles' tendinosis surgery)	Tendinopathy	17	6 M<br>11 F	27-68
	Control (healthy individuals)	No Tendinopathy	4	4 M	21- 48
4. Dean et al 2015	Case (subacromial decompression surgery)	Tendinopathy	9	7M<br>2F	51 +/- 8.2
	Control (5-years after subacromial decompression ('pain-free')	No Tendinopathy	9	6M<br>3F	52 /- 7.8
5. Fearon et al 2014	Case (gluteal tendon reconstructive surgery and bursectomy)	Severe Tendinopathy (SD 12.65)	34	-	-
	Control (total hip arthroplasty)	Mild Tendinopathy (SD 10.43)	29	-	-

Study	Group	Tendon Status	Sample Size	Sex	Age, years (mean and/or range)
6. Franklin et al 2014	Case (arthroscopic or open tendon repair)	Tendinopathy	64	39 M<br>25 F	50- 78
	Control (post-traumatic shoulder instability)	No Tendinopathy	16	14 M<br>2 F	17-29
7. Sasaki et. al., 2013	Case (recalcitrant tennis elbow)	Tendinopathy	8	2 M<br>6 F	38-66
	Control (ECRB capsule of Osteochondritis Dissecance of Capitellum)	No Tendinopathy	2	1 M<br>1 F	15
8. Tosounidis et al 2013	Case: RC tear and biceps tendinitis	Tendinopathy	14	6 F<br>8 M	51-76
	Control A (shoulder hemiarthroplasty for management of complex proximal humerus fractures)	No Tendinopathy	17	1 M<br>16 F	56-81
	Control B (specimens from cadavers with no history of shoulder pain, trauma or systemic disease)	No Tendinopathy	10	2 M<br>8 F	60- 82
9. Schizas et al 2012	Case (jumper's knee)	Tendinopathy	10	9 M<br>1 F	19-32
	Control (tibial shaft fractures undergoing intramedullary nailing)	No Tendinopathy	8	5 M<br>3 F	19-60

Study	Group	Tendon Status	Sample Size	Sex	Age, years (mean and/or range)
10. Bagge et al 2012	Case (Achilles' tendinosis surgery)	Tendinopathy	2	1 M<br>1 F	29<br>52
	Control	No Tendinopathy	2	2 F	47
11. Bjorklund et al 2011	Case (Achilles' tendinosis surgery)	Tendinopathy	17	8 M<br>9 F	28-70<br>47-68
	Control (healthy tendon)	No Tendinopathy	7	3 M<br>4 F	39-46<br>21-47
12. Xu et al 2011	Case (rotator cuff tear repair)	Tendinopathy	26	14 M<br>12 F	30-73
	Control (shoulder instability)	No Tendinopathy	10	3 F<br>7 M	17-59
13. Schizas et al 2010	Case ((jumper's knee)	Tendinopathy	10	9 M<br>1 F	19-32
	Control (tibia fractures- intramedullary nailing without current or previous knee pain)	No Tendinopathy	8	5 M<br>3 F	16-53
14. Bagge et al 2009	Case (Achilles' tendinosis)	Tendinopathy	15	9 M<br>6 F	23-59
	Control (healthy tendon)	No Tendinopathy	5	2 M<br>3 F	39-47

**Study**	**Group**	**Tendon Status**	**Sample Size**	**Sex**	**Age, years (mean and/or range)**
15. Bjur et al 2009	Case (Achilles' tendinosis surgery)	Tendinopathy	37	17 M<br>20 F	26-61
	Control (pain-free Achilles' tendons)	No Tendinopathy	8	3 M<br>5 F	21-47
16. Zeisiget al 2009	Case (tennis elbow surgery & golfer's elbow surgery)	Tendinopathy	7 & 4	4 M<br>3 F &<br>2 M<br>2 F	32-52<br><br>24-40
	Control (pain free healthy individuals)	No Tendinopathy	6	5 M<br>1 F	24-40
17. Singaraju et al 2008	Case (arthroscopically assisted biceps tenodesis)	Tendinopathy and tenosynovitis	6	3 M<br>3 F	44-60
	Control (healthy cadavers)	No Tendinopathy and tenosynovitis	6	5 M<br>1 F	42-81
18. Andersson et al 2008	Case (IF; chronic painful mid-portion Achilles tendinosis)	Tendinopathy	20	9 M<br>11F	26-67
	Control (IF; healthy pain free Achilles' tendons)	No Tendinopathy	7	4 M<br>3 F	33-46
	Case (ISH; chronic painful mid-portion Achilles tendinosis)	Tendinopathy	9	3 M<br>6 F	37-56

Study	Group	Tendon Status	Sample Size	Sex	Age, years (mean and/or range)
	Control (ISH; healthy pain free Achilles' tendons)	No Tendinopathy	3	3	47
19. Bjur et al 2008	Case (IF; chronic painful mid-portion Achilles tendinosis)	Tendinopathy	21	8 M<br>13 F	43 (mean age)<br>47 (mean age)
	Control (IF; healthy pain free Achilles' tendons)	No Tendinopathy	8	4 M<br>4 F	37 (mean age)<br>40 (mean age)
	Case (ISH; chronic painful mid-portion Achilles tendinosis)	Tendinopathy	2		
	Control (ISH; healthy pain free Achilles' tendons)	No Tendinopathy	1		
20. Scott et al 2007	Case (Patellar + Achilles tendinopathy)	Tendinopathy	1 + 13	19 M	18-54
	Control (healthy pain free individuals)	No Tendinopathy	8 + 7	10 F	
21. Danielson et al 2007 (1)	Case (chronic painful tendinosis)	Tendinopathy	2	1 M<br>1 F	22<br>23
	Control (pain-free patellar tendon)	No Tendinopathy	1	1 M	22
22. Danielson et al 2007 (2)	Case (unspecified surgical treatment)	Tendinopathy	7	6 M	22-32

Study	Group	Tendon Status	Sample Size	Sex	Age, years (mean and/or range)
				1 F	
	Control (normal control tissue- skin incision)	No tendinopathy	15	14 M<br>1 F	20-47
23. Danielson et al 2006	Case (proximal patellar tendinopathy)	Tendinopathy	7	6M<br>1F	27 (22-32)
	Control (pain-free and normal patellar tendons)	No Tendinopathy	16	15M<br>1F	32.1, 20-47
24. Lian et al 2006	Case (jumper's knee)	Tendinopathy	10		24-34
	Control (tibia fracture undergoing marrow nailing)	No Tendinopathy	10		19-43
25. Bjur et al 2005	Case (chronic painful mid-portion Achilles' tendinosis)	Tendinopathy	21	8 M<br>13 F	35-54<br>34-56
	Control (normal Achilles' tendons)	No Tendinopathy	9	4 M<br>5 F	35-60<br>22-46
26. Forsgren et al 2005	Case (Achilles' & Patellar tendinosis)	Tendinopathy	6<br>12		
	Control (normal tendons)	No Tendinopathy	13<br>5		
27. Alfredson et al 2001	Case (chronic painful Achilles tendinosis)	Tendinopathy	9	3 M<br>6 F	45 (mean)

Study	Group	Tendon Status	Sample Size	Sex	Age, years (mean and/or range)
	Control (normal (pain-free) Achilles tendons)	No Tendinosis	2	1 M<br>1 F	39
28. Alfredson et al 2000 (1)	Case ((Jumper's knee)	Tendinopathy	5	4 M<br>1 F	23-31
	Control (healthy tendon)	No Tendinopathy	5	4 M<br>1 F	27-43
29. Alfredson et al 2000 (2)	Case (surgical treatment of tennis elbow)	Tennis Elbow	3	3 M<br>1 F	29-54
	Control (painful elbow)	No tendinopathy	2	2 F	28-43
30. Alfredson et al 1999	Case (Achilles tendinosis)	Tendinopathy	4	4M	40.7, 34-53
	Control (healthy tendon)	No Tendinopathy	5	5M	37.2, 27-42
31. Ljung et al 1999	Case (tennis elbow)	Tendinopathy	6	3 M<br>3 F	38-52
	Control (healthy tendon)	No tendinopathy	6	5 M<br>1 F	24-39

Supplementary Table 3: Study Characteristics – part B

No.	Study	Result
**1.**	Schmalzl J et al., 2019	NSE immunohistochemical staining observed high density of free nerve endings at the transition zone to the paratenon in inflamed tendons compared to the no tendinitis group.
**2.**	Sahmey et al 2016	a) CGRP, PGP9.5 (a neuroendocrine marker) and SP immunoreactions also occurred in abnormal chondrocyte-like cells.<br>b) SP-labelled fibres, more intimately associated with vessels, were only detected in some tendinopathic tendon<br>c) a greater expression of SP in tendons that exhibited immature vessels.<br>d) Synaptophysin-immunoreactive nerves were closely related to vessels in tendinopathy
**3.**	Christensen J et al 2015	a) Double staining of the PAR receptors and SP showed that nerve fibres and fascicles expressing the PAR-receptors often co-localised with SP, however not all the nerve fibres expressing PARs were positive for SP.<br>b) Protease activated receptors are expressed in the Achilles tendon and surrounding tissues<br>- PAR 1 and 4 predominantly in nerves, whilst PAR-2 by tenocytes<br>- all 4 PAR receptors colocalised with SP positive nerve fibres
**4.**	Dean et al 2015	- Results<br>o No difference in glutamate between groups (p=.86)<br>o No difference in NMAR1 between groups (p=.61)<br>o Significantly higher PGP-9.5 in painful group (3.75 vs 0.87) (p=.0079)<br>o Significantly higher mGluR2 in painful group (0.064 vs 0.0019) (p=.05)<br>o No difference in mGluR1<br>o Significantly higher mGluR7 in pain-free group (0.18 vs 0.005) (p=.0019)<br>o Significantly higher Kainate receptor 1 (KA1) in painful group (4.55 vs 0.85) (p=.0028)<br>o *mGluR = metabotrophic glutamate receptor<br>o Correlations<br>▪ No strong correlation between PGP-9.5 & glutamate receptor expression<br>▪ No strong correlation between PGP-9.5 & TNF-alpha expression<br>o Explored other inflammatory cells (macrophages etc), but doesn't seem as relevant
**5.**	Fearon et al 2014	- significantly greater presence of SP in the bursa but not in the tendon (p-.223) of subjects with GTPS vs controls<br>- SP was expressed in fibroblast like cells embedded within the bursa stroma or within the tendon and in close association with vessels both in bursa and in tendon
**6.**	Franklin et al 2014	a) Glutamate → P < 0.001 tear vs control<br>b) NMDAR1 → P < 0.001 tear vs control<br>c) mGluR2 → P=.008 overexpressed in tears vs control<br>d) mGlur7 → dramatically reduced (P<0.001)<br>e) mGlur8 → significant in small tears vs large/medium tears (1-3 cm tears)<br>f) NK-1 → lower vs control (p=0.007)<br>g) BDKRB2 → reduced in tears (p=0.354)<br>h) PGP9.5 → significant difference between small tear and large tear (p=.021)

		i) Nav1.7 → no significant difference<br>j) TRPA1 → significantly reduced in small tears vs large ones (p=.001)<br>k) No changes in SP and CGRP expression<br>l) Increased expression of alpha-2a adrenergic receptors<br>m) TH → reduced (p=.0235)
**7.**	Sasaki et. al., 2013	a) intensity of the PGP 9.5 and NPY was stronger in the tendinosis tissue compared to control tissue.<br>b) decreased immunoreactivity of CGRP and SP in tendinosis tissue.<br>c) increased sympathetic innervations + loss of sensory innervations of the tendinosis tissue at the ECRB capsule<br>d) perivascular sensory innervation was limited in the tendinosis tissue whilst there were marked immunoreactions for sympathetic nerve markers
**8.**	Tosounidis et al 2013	a) showed S-100 and NPY, adrenergic in 11/14 cases of RC tear and biceps tendinitis<br>b) Alpha 1 adrenergic immunoreactions were positive in a subpopulation of cells that expressed NPY
**9.**	Schizas et al 2012	a) increased tissue immunodensity of NMDAR1, phosphor-NMDAR1 and mGluR5, SP vs control<br>b) NMDAR1 predominant in peritendinous tissue whilst phospho-NMDAR1 in tendon proper<br>c) presence of sprouting nerve fibres in tendon proper (Positive PGP 9.5 staining)<br>d) mGlur5 distinctive of late-stage tendinopathy, predominant on altered tenocytes and free nerve fibres in tendinopathy biopsies<br>e) SP present on both peritendinous and tendon proper tissue<br>f) SP on sprouting nerve fibres in 5 out 10 biopsies exhibiting signs of late stage tendinosis vs absent in control<br>g) the occurrence and immunodensity of NMDAR1 correlated with that of SP in tendinopathic samples vs not so in control<br>h) co-localisation between NMDAR1 and SP and phosphor-NMDAR1 and SP both in the tendinopathic and control biopsies, however only tendinopathic biopsies exhibited co-localisation of SP and phosphor-NMDAR1 within the tendon proper.
**10**	Bagge et al 2012	- ISH results-<br>- tendinosis tenocytes showed specific BDNF mRNA reaction<br>- specific mRNA reactions were noted for tenocytes in non-tendinosis patients<br>- IHC results-<br>- large number of tenocytes showed BDNF immunoreactivity in both tendinosis and non-tendinosis groups<br>- BDNF is produced in the tenocytes of the human Achilles tendon, however BDNF immunolabelling and BDNF mRNA is not confined to all tenocytes in the Achilles tendon
**11**	Bjorklund et al 2011	- IHC-F results<br>a) Difference in CB1 expression between groups was statistically significant (P<.05) with it being higher in the tendinosis group vs control
**12**	Xu et al 2011	- IHC results<br>a) Immunoreactivity for PGP9.5 and GAP43 was rarely seen in the tendon tissue proper, but rather in the paratendinous tissue and endotenon between collagen bundles and near blood vessels

		b) Large groups of nerve fascicles observed in torn and matched tendon groups vs control<br>- GAP43 and PGP9.5 observed within tendon proper and or intimately associated with blood vessels<br>c) Quantitative analysis showed that number of PGP9.5 and GAP43 immunoreactive nerves were significantly higher in matched subscapularis tendons vs control subscapularis tendons (P<0.05) and torn supraspinatus tendons (P<.0002 and P<.0001).
**13**	Schizas et al 2010	Quantitative Assessment<br>1) **NMDAR1**<br>a) No vessels within the tendon proper,<br>b) vascular **NMDAR1** in the tendon proper (5.9%) of tendinopathic group exhibited similar levels as in the paratendinous tissue<br>c) 9 fold increase in vascular NMDAR 1 in tendinopathic tenocytes vs control<br>**2) Glutamate**<br>a) Glutamate occurrence in vessels and cells was elevated 10 times in tendinopathic group<br>b) Increased number of glutamate positive tenocytes in tendinopathic tendons vs control (p= 0.009)<br>c) Vascular glutamate localised in the paratendinous tissue higher vs control<br>3) **Correlation of NMDAR1 and glutamate occurrence**: no correlation found in either 2 groups<br>Combined IF and DAB staining<br>1) NMDAR1<br>a) Increased NMDAR1 immunoreaction in painful tendons, localisation of increased NMDAR1 immunostaining in tendinopathic samples<br>b) Occurrence of NMDAR1 with PGP9.5- elevated in tendinopathy vs control<br>2) Glutamate<br>a) Glutamate was elevated in tendinopathic tissue vs control<br>b) Occurrence of glutamate with PGP9.5- in tendinopathy vs control<br>c) Increased glutamate positive tenocytes in tendinopathic tissue vs control<br>3) Co-localisation of NMDAR1 and glutamate:<br>a) Demonstrated in nerve fibres in all tendinopathic tissues vs none in control
**14**	Bagge et al 2009	There are marked immunoreactions for the neurotrophins NGF and BDNF and for the p75 receptor, but not for TrkA or TrkB, in the tenocytes of the human Achilles tendon
**15**	Bjur et al 2009	a) NPY- immunoreactions were seen in the nerve fascicles, and mildly in the perivascular nerve fibres, but none in the tenocytes.<br>b) Y1 receptor- immunoreactions present in both non-tendinosis and tendinosis groups, seen in tenocytes and blood vessel walls<br>- stronger immunoreactions present in tendinosis group vs control (p<0.01)<br>c) Y2 Receptor- no immunoreactions in blood vessels wakks, tenocytes or nerve fascicles.<br>-
**16**	Zeisiget al 2009	- showed presence of catecholamine-synthesising enzyme TH in the fibroblasts of the tissue samples from 4/7 patients with TE and 2/4 patients with GE, and no detectable levels of this enzyme were found in fibroblasts of control tissue from the lateral epicondyle (0/6).<br>- no evidence of such production in patients with TE or GE was found in the present study using staining for the ACh-synthesising enzyme ChAT.<br>- no evidence of nerves positive for ChAT, whereas several nerve structures displaying TH-immunohistochemical reactions were detected.
**17**	Singaraju et al 2008	The IHC staining detecting CGRP and substance P was found globally throughout the tendon body in the proximal and distal sections of both groups with no significant differences between the control and experimental tendons.

**18**	Andersson et al 2008	- The nerve fascicles were seen to contain nerve fibers showing SP-immunoreactions.<br>- The results of the present study show that tenocytes of Achilles tendons display expression of SP and NK-1 R.<br>- Expression at both protein and mRNA levels was shown for the NK-1 R, whilst SP was demonstrated at the mRNA level.<br>- The labelling was detected for a subpopulation of the tenocytes, the semi-quantitative estimations suggesting higher expression levels of both NK-1 R and SP in tendinosis tendons compared with normal tendons.
**19**	Bjur et al 2008	- TH and NPY innervation perivascularly in both control and tendinosis tendons for both paratendinous CT and tendon tissue proper<br>- Distinct occurrence of alpha 1 adrenoreceptors including in tenoyctes in tendinosis specimens<br>- Tendinopathic tenocytes had the occurrence of TH-LI<br>- TH immunoreactions were more common in tenocytes than nerves<br>- TH mRNA- ISH reactions were observed for tenocytes<br>- Limited sympathetic innervation but abundant adrenoceptors
**20**	Scott et al 2007	IHC-F results<br>- VGlut1- no immunoreactions in tendon<br>- VGlut2 immunofluorescence was observed in tendon- in tenocytes<br>- Semiquantitative grading revealed a significantly greater expression of VGLuT2 in tenocytes from tendinosis patients than in those of controls (p=.005).<br>In situ Hybridisation<br>- VGluT2 mRNA expression in tenocytes
**21**	Danielson et al 2007 (1)	ISH results<br>- Tenocytes of the tendinosis specimens displayed a stronger and more frequent AP reactions vs control<br>- Occurrence of mRNA for TH in tenocytes is positive
22	Danielson et al 2007 (2)	- IHC results: General and Sensory Innervation Patterns<br>1) PGP 9.5- specific reactions for PGP 9.5 abundant in areas of loose CT<br>2) SP/ CGRP or SP-CGRP-LI were overall rarely detected in specimens of the tendinosis tendons; this corresponded to the normal tendon tissue proper<br>3) Sympathetic innervation patterns<br>- Normal tendons: lower NPY and TH immunoreactive nerve fibres than in the loose paratendinous CT<br>- Tendinosis Tendons: NPY & TH immunoreactive nerve fibres abundant in loose CT and around blood vessels<br>- not a lot of difference btw normal and tendinosis samples<br>4) Adrenergic Receptors<br>- nerve fascicles in the tendinous tissue displayed an immunoreaction pattern for adrenergic receptors similar to that of the loose paratendinous CT of controls, with greatest immunoreaction being for alpha 1 adrenoreceptor<br>- alpha-1 adrenoreceptors in tendinosis tendon vasculature was more marked vs control by semiquantitative analysis<br>- limited immunoreactions for alpha 2A adrenoreceptors in both healthy and tendinosis tissue

		- no general difference in the occurrence of beta1-adrenoreceptor in tendinosis vs control<br>- tenocytes exhibited adrenergic receptor LI, alpha 2A adrenoreceptor-LI with more distinct immunoreaction compared to control<br>5) TH-like immunoreactions (-LI) in tenocytes<br>- immunoreactions more distinct in tendinosis tendon samples vs normal<br>6) The amount of sympathetic innervation did not match the quantity of adrenergic receptors in the tendon tissue proper of the patellar tendon, particularly in tendinosis.<br>- These findings suggest that locally produced catecholamines can be mediators that bind to the frequently occurring adrenergic receptors
23	Danielson et al 2006	- Results<br>○ M2 receptor<br>▪ Immunoreaction in blood vessel walls observed in both groups, more pronounced in tendinosis group particularly in those with hyper cellularity<br>▪ No immunoreaction in tenocytes & nerve fascicles observed in controls<br>▪ Immunoreaction in tenocytes & nerve fascicles observed in specimens, particularly in those with hypercellularity<br>○ Choline acetyltransferase (ChAT)<br>▪ No immunoreaction in blood vessels or tenocytes in control<br>▪ Immunoreaction in blood vessels observed in tendinosis, particularly in those with profound hyper cellularity or abnormally appearing tenocytes<br>▪ No immunoreaction in tenocytes in specimens<br>○ Vesicular Acetylcholine transporter (VAChT)<br>▪ No immunoreaction in blood vessels or tenocytes in control<br>▪ Some immunoreaction in tenocytes seen in specimens, more so in those with hypercellularity or with abnormal appearance<br>▪ No immunoreaction in blood vessels in specimens<br>○ Acetylcholinesterase (AChE)<br>Immunoreaction observed in blood vessels & tenocytes of both groups, with no convincing differences between both groups
24	Lian et al 2006	Semiquantitative Analysis tendon vs control<br>- higher occurrence of SP= 0.567<br>- higher occurrence of PGP= 0.098<br>- lower occurrence of TH = 0.018
25	Bjur et al 2005	IHC results<br>a) Innervation patterns- PGP9.5 was seen in tendinosis tissue, the staining was seen intimately associated with fine blood vessels unlike control<br>b) Immunoreactions against CPRP and SP were also detected in thin nerve fascicles and as freely coursing nerve fibres, sometimes being closely located to fine blood vessels<br>c) In normal tendon specimens, the immunoreaction for CGRP was more marked than that for SP<br>d) CGRP/SP immunoreaction was only observed in the association with a subpopulation of the blood vessels

26	Forsgren et al 2005	- A variety of NK-1R antibodies were used<br>- Results (pretty rubbish in my opinion).<br>○ NK-1R immunoreaction found in blood vessel walls (greater extent) in both groups<br>○ NK-1R immunoreaction found in nerve fibers/ fascicles (lesser extent) in both groups<br>NK-IR immunoreaction occurred to various extents in both tendinosis groups, with greater presence in tendinosis specimens with pronounced vascularization.
27	Alfredson et al 2001	Results (Achilles tendon)<br>- Micro dialysis- presence of free glutamate in all tendons<br>a) Tendinosis- 78-250umol/l<br>b) No tendinosis- 16-34umol/l<br>- NMDAR1 receptor detected in all tissues<br>AChE and NMDAR1 reactions often localised to similar structures
28	Alfredson et al 2000 (1)	Results (Patellar tendon) Microdialysis- HPLC and IHC:<br>- The mean concentration of glutamate was significantly higher than the mean concentration for glutamate in control<br>- No significant differences between the mean conc of PGE2 in tendonosis vs control<br>Glutamate NMDAR1 receptors present in all tendons (localised to AChE structures)
29	Alfredson et al 2000 (2)	Results: Microdialysis<br>ECRB tendons had higher conc of glutamate vs control ($p<.001$)
30	Alfredson et al 1999	- Microdialysis results<br>○ Glutamate concentration was significantly higher in tendinosis (196 ± 59 μmol/l) vs controls (48 ± 27 μmol/l) across all timepoints over 4hr period ($p<.05$)<br>○ No significant difference in mean concentrations of glutamate over 4hrs between 2 groups<br>- No significant difference in PGE2 or mean PGE2 between 2 groups
31	Ljung et al 1999	- A quantitative analysis of the vessels and nerves in patients with tennis elbow compared to those in control was not possible.<br>- The extensor carpi radialis brevis muscle is supplied with SP and CGPR

BMJ Publishing Group Limited (BMJ) disclaims all liability and responsibility arising from any reliance placed on this supplemental material which has been supplied by the author(s)

Supplementary Table 4: A summary of the most important findings of each study.

Article No.	SP	CGRP	NMDAR Receptors	Glutamate	Glutamate Receptors mGlut	PGP 9.5	NK-1R	Tyrosine hydroxylase	Neuropeptide-Y (NPY)	NPY Receptors	AChE	Adrenoreceptors	Others
**1. Schmalzl J et al., 2019**													Neuron-Specific Enolase (NSE)
**2. Sahmey et al 2016**	✓	✓				✓							
**3. Christensen et al., 2015**	✓												PAR receptors
**4. Dean et al 2016**			✓	✓	✓	✓							KA1
**5. Fearon et al 2014**	✓												
**6. Franklin et al 2014**	✓	✓	✓		✓	✓	✓	✓				✓	Nav1.7<br>TRPA1 BDKRB2
**7. Sasaki et al 2013**	✓	✓				✓			✓				
**8. Tosounidis et al 2013**									✓			✓	S-100
**9. Schizas et al 2012**	✓		✓		✓	✓							
**10. Bagge et al 2012**													BDNF
**11. Bjorklund et al 2011**													CB1
**12. Xu et al 2011**						✓							GAP 43
**13. Schizas et al 2010**			✓	✓									
**14. Bagge et al 2009**													NGF<br>BDNF<br>P75
**15. Bjur et al 2009**									✓	✓			
**16. Zeisiget et al 2009**								✓					
**17. Singaraj et al 2008**	✓	✓											
**18. Andersson et al 2008**	✓						✓						
**19. Bjur et al 2008**								✓	✓			✓	
**20. Scott et al 2007**					✓								
**21. Danielson et al 2007 (1)**								✓					

**22. Danielson et al 2007 (2)**	✓	✓				✓		✓	✓			✓	
**23. Danielson et al 2006**											✓		M2 Ach Receptor ChAT, VAChT
**24. Lian et al 2006**	✓					✓		✓					
**25. Bjur et al 2005**	✓	✓				✓							
**26. Forsgren et al 2005**							✓						
**27. Alfredson et al 2001**			✓	✓							✓		
**28. Alfredson et al 2000 (1)**			✓	✓							✓		
**29. Alfredson et al 2000 (2)**				✓									
**30. Alfredson et al 1999**				✓									
**31. Ljung et al 1999**	✓	✓											

Supplementary Table 5: Markers of neurogenic inflammation assessed in each study

**High Quality (Score: >12)**	**Quality Assessment Score**	**Moderate Quality (Score: 10-12)**	**Quality Assessment Score**	**Low Quality (Score: <10)**	**Quality Assessment Score**
	11	Sahemey R et. al, 2016)	10	Schmalzl et. al, 2019	9
Dean B. J. et al, 2015	15	Zeisig E et. al, 2009	11	Bagge J et. al, 2009	6
Franklin S. L. et al, 2014	14	Lian O et. al, 2006	11	Bjur D, et. al, 2009	9
Fearon A. M. et al, 2014	15	Sasaki K. et. al, 2013	12	Andersson G; et. al, 2008	4
Tosounidis T. et al, 2013	15	Bagge et. al, 2012	10	Bjur D et. al, 2008	6
Xu Y et. al 2011	13	Schizas N. et. al, 2010	10	Scott A et. al, 2007	9
		Singaraju V. M. et. al, 2008	10	Danielson P et. al, 2007 (1)	7
		Christensen J et al, 2015	11	Danielson P et. al, 2007 (2)	7
		Schizas et al, 2012	11	Danielson, P, et. al, 2006	7
		Bjorklund et. al, 2011	11	Bjur D et. al, 2005	7
				Forsgren, S et. al, 2005	5
				Alfredson H et. al, 2001	5
				Alfredson H et. al, 2000 (1)	9
				Alfredson, et. al, 2000 (2)	9
				Ljung B. O et. al, 1999	6
				Alfredson, H. et. al, 1999	8

Supplementary table 6. Results of study quality assessment
